# Efficacy and Safety of Daratumumab‐Based Regimens in Multiple Myeloma: A Systematic Review and Meta‐Analysis of Phase III Randomized Controlled Trials

**DOI:** 10.1002/jha2.70362

**Published:** 2026-07-25

**Authors:** Abdul Moiz Khan, Hafsa Ajmal, Muddassir Khalid, Waleed Babar, Usama Shafaqat, Tooba Fida, Mohammad Aitzaz Hassan, Sibgha Fatima, Ahmad Sameed Akram, Fred Segawa

**Affiliations:** ^1^ Mayo Clinic Jacksonville Florida USA; ^2^ Department of Medicine King Edward Medical University Lahore Pakistan; ^3^ Department of Medicine Nishtar Medical University Multan Pakistan; ^4^ Department of Medicine Allama Iqbal Medical College Lahore Pakistan; ^5^ Department of Medicine ISMMS/Valley Health Paramus Paramus USA; ^6^ Department of Medicine Gomal Medical College D.I. Khan Pakistan; ^7^ Department of Medicine Makerere University College of Health Sciences Kampala Uganda

**Keywords:** daratumumab, malignancy, multiple myeloma, progression‐free survival, relapsed/refractory multiple myeloma

## Abstract

**Introduction:**

Multiple myeloma is a malignant plasma cell disorder predominantly affecting older adults and poses a significant health burden due to its incurable nature, high relapse rate, and associated immune dysfunction. Daratumumab, when added to standard therapy, has shown promising results in large trials. This meta‐analysis compared clinical trials of daratumumab‐containing regimens with standard therapies in the treatment of multiple myeloma. The primary outcomes analyzed were overall response rate (ORR) and progression‐free survival (PFS).

**Methods:**

A systematic search of PubMed, Scopus, Cochrane CENTRAL, and Google Scholar was performed from January 2015 through December 2024. After screening via Rayyan, data were extracted on an Excel spreadsheet. Quality was assessed using the Cochrane RoB 2.0 tool. Eligible randomized controlled trials (RCTs) were included and analyzed using RevMan 5.4. Outcomes assessed were ORR and PFS. Adverse effects were compared among therapies.

**Results:**

Nine Phase III RCTs (*n* = 4556) were included, with a mean follow‐up of 44.3 months. Daratumumab‐based regimens significantly improved PFS (HR 0.55, 95% CI 0.46–0.66) but did not confer a statistically significant overall survival advantage (RR 0.90, *p* = 0.25). ORR was inconsistent, with five trials favoring daratumumab and one favoring control. Safety analyses showed higher risks of pneumonia (RR 1.74, *p* < 0.0001), secondary malignancies (RR 1.46, *p* = 0.003), neutropenia (RR 1.25, *p* = 0.006), and thrombocytopenia (RR 1.17, *p* = 0.01). Lymphopenia showed a numerical increase but was not statistically significant.

**Conclusion:**

Daratumumab plus standard therapy regimens significantly improve PFS in multiple myeloma, though the overall survival benefit remains unproven. While efficacy is notable, treatment is associated with increased risks of infections, hematological toxicities, and secondary malignancies, highlighting the need for careful patient selection and vigilant monitoring.

**Trial Registration:**

The authors have confirmed clinical trial registration is not needed for this submission.

## 1 Introduction

Multiple myeloma is a malignant blood disease characterized by the uncontrolled proliferation of plasma cells, resulting in the production of nonfunctional immunoglobulins. It accounts for about 1% of all cancers worldwide and 10%–15% of hematological malignancies [1]. It usually affects older adults with a median age at diagnosis of 69 years. Each year, about 35,000 people in the US and about 588,000 worldwide are diagnosed with multiple myeloma. People with multiple myeloma are at an increased risk of infection due to immune dysfunction [2]. Multiple myeloma has a lifetime risk of 0.7% among the general population. It poses a substantial burden both on the healthcare system as well as patients. Multiple myeloma has no cure. Despite treatment, the majority of the patients eventually experience relapse or disease progression [3]. Over past decades, drugs such as proteasome inhibitors and immunomodulators have contributed to improvement in survival of patients with multiple myeloma. However, the outcomes of the patients with relapsed myeloma implies the need for newer therapies. Daratumumab is a monoclonal antibody that targets CD38, a transmembrane glycoprotein, expressed at constantly high levels by myeloma cells and thus an attractive target for therapy [4]. The Food and Drug Agency (FDA) and European Medicines Agency (EMA) approved daratumumab as monotherapy for multiple myeloma in 2015 and 2016 respectively [5, 6]. According to a meta‐analysis, since daratumumab's approval, its incorporation into myeloma treatment regimens has led to significant improvement in treatment outcomes such as stringent complete response (sCR) and minimal residual disease (MRD) negativity. This has resulted in prolonged progression‐free survival (PFS) and overall survival (OS) [6]. Recent meta‐analysis demonstrated the efficacy and safety of daratumumab in treatment of RRMM (Refractory/Relapsed Multiple Myeloma) [7]. Although previous meta‐analyses have evaluated daratumumab in relapsed/refractory multiple myeloma, several recently published Phase III trials have expanded its use into newly diagnosed and transplant‐eligible populations. Consequently, an updated synthesis incorporating these contemporary studies is needed to provide a more comprehensive assessment of the efficacy and safety of daratumumab across the broader multiple myeloma treatment landscape.

## 2 Methodology

This study was conducted in accordance with the PRISMA 2020 guidelines.

### 2.1 Data Sources and Search Strategy

A systematic search of PubMed, Scopus, Cochrane CENTRAL, and Google Scholar was performed from January 2015 through December 2024. The search strategy employed a combination of keywords including “daratumumab,” “multiple myeloma,” and “progression‐free survival.” After removing duplicate entries, the remaining records were screened based on their titles and abstracts. Full texts of potentially relevant studies were then retrieved and assessed for eligibility in accordance with predefined inclusion and exclusion criteria.

### 2.2 Study Selection

**FIGURE 1:**
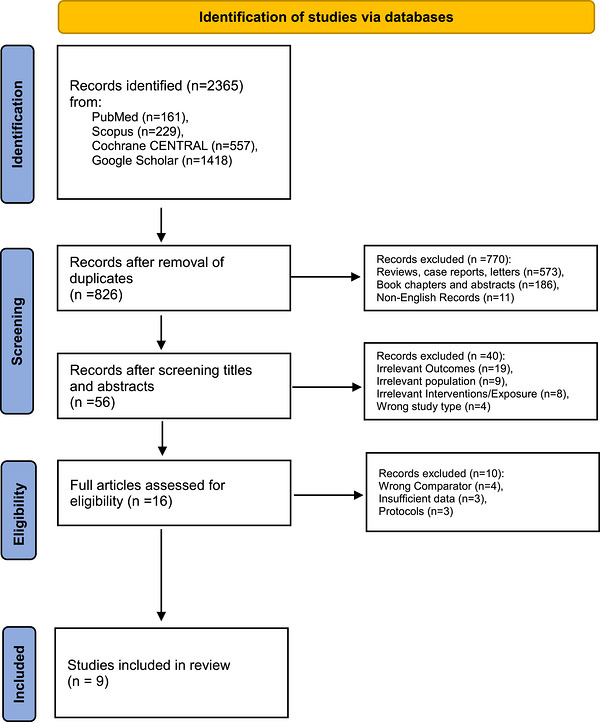
PRISMA Chart.

Studies were included if they met the following criteria: Publication in English with freely accessible full‐text availability; completed and published randomized controlled trials (RCTs) of Phase 3 or higher; inclusion of daratumumab as an intervention either alone or in combination with standard therapy; a comparator group without daratumumab; and PFS designated as the primary endpoint. Studies were excluded if they were cohort or case‐control in design, did not use daratumumab as part of the intervention, or failed to report PFS as a primary outcome. After applying these criteria, a total of nine RCTs were deemed eligible for inclusion in the final analysis (Figure 1).

### 2.3 Data Extraction

Data extraction was independently conducted by two authors, who collected the following information from each included study: Name of the first author, year of publication, disease status (relapsed/refractory or not), treatment regimens, sample size for both daratumumab and control groups, median age, number of prior therapy lines, follow‐up duration, dose of daratumumab (in mg), and study outcomes. Outcomes included PFS, overall response rate (ORR), and adverse events such as pneumonia, malignancy, neutropenia, lymphopenia, thrombocytopenia, and mortality. Upon completion of data extraction, the data were compiled for statistical analysis and summarized in Table 1.

**TABLE 1:** Baseline Characteristics of included trials.

Name of study	Sonneveld P. (PERSEUS)	Dimopoulos MA et al. (POLLUX)	Usmani SZ. et al. (CANDOR)	Fu W. et al. (OCTANS)	Moreau P. et al. (CASSIOPEIA)	Sonneveld et al.2022 (CASTOR)	Meletios A Dimopoulos et al. (APOLLO)	Maria‐Victoria et al. (ALCYONE)	Weijun Fu et al. (LEPUS)
Study design	Randomized phase III trial	Randomized phase III trial	Randomized phase III trial	Randomized phase III trial	Randomized phase III trial	Randomized phase III trial	Randomized phase III trial	Randomized phase III trial	Randomized phase III trial
Year	2024	2018	2023	2023	2021	2022	2023	2025	2023
Relapse/refractory disease	Yes	Yes	Yes, Relapsed and refractory multiple myeloma	No	No	yes, Relapsed and refractory multiple myeloma	yes, Relapsed and refractory multiple myeloma	No	yes, Relapsed and refractory multiple myeloma
Regimen	Daratumumab, Bortezomib, Lenalidomide and Dexamethasone vs. Bortezomib, Lenalidomide and Dexamethasone	Daratumumab, Lidomide and Dexamethasone vs. Lidomide + Dexamethasone	Carfilzomib, Dexamethasone, Daratumumab vs. Carfilzomib, Dexamethasone	Daratumumab plus bortezomib and dexamethasone (D‐Vd) vs. bortezomib and dexamethasone (Vd) alone	part 1=D‐VTd (daratumumab, bortezomib, thalidomide dexamethasone) vs. VTd part 2 = Daratumumab vs. observation only	Daratumumab plus bortezomib and dexamethasone (D‐Vd) vs bortezomib and dexamethasone (Vd) alone	Daratumumab plus pomalidomide and dexamethasone vs Pomalidomide and dexamethasone	Daratumumab plus bortezomib, melphalan, and prednisone (D‐VMP) vs. bortezomib, melphalan, and prednisone (VMP) alone	Daratumumab plus bortezomib/dexamethasone (D‐Vd) vs. Bortezomib/dexamethasone (Vd)
No. of patients	709	304	466	220	886	498	304	706	211
Daratumumab	355	151	312	146	442	251	151	350	141
Control	354	153	154	74	444	247	153	356	70
Mean age	60	67	64	69	59	64	67	71	61
Prior lines of therapy, median (range)	Not eligible	all 304 patients (Pis, IMDs and anti CD38)	bortezomib, lenalidomide, pomalidomide	Corticosteroids	No prior therapy	2 (1–10) bortezomib (65.5%), thalidomide (49.4%), lenalidomide (42.0%), and both a protease inhibitor and immunomodulatory drug (48.4%).	lenalidomide + a proteasome inhibitor, and refractory to lenalidomide if only one prior line	No prior therapy	79.1% of patients previously received bortezomib, 26.5% were refractory to lenalidomide.
Follow up duration (months)	47.5	39.1	50	12.3	35.4	72.6	39.6	86.7	25.1
Dose of daratumumab (mg)	16 mg/kg (1800 mg)	16 mg/kg	8 mg/kg (first 2 days), then 16 mg/kg	16 mg/kg	16 mg/kg	16 mg/kg	16 mg/kg	16 mg/kg	16 mg/kg

### 2.4 Quality Assessment

**FIGURE 2:**
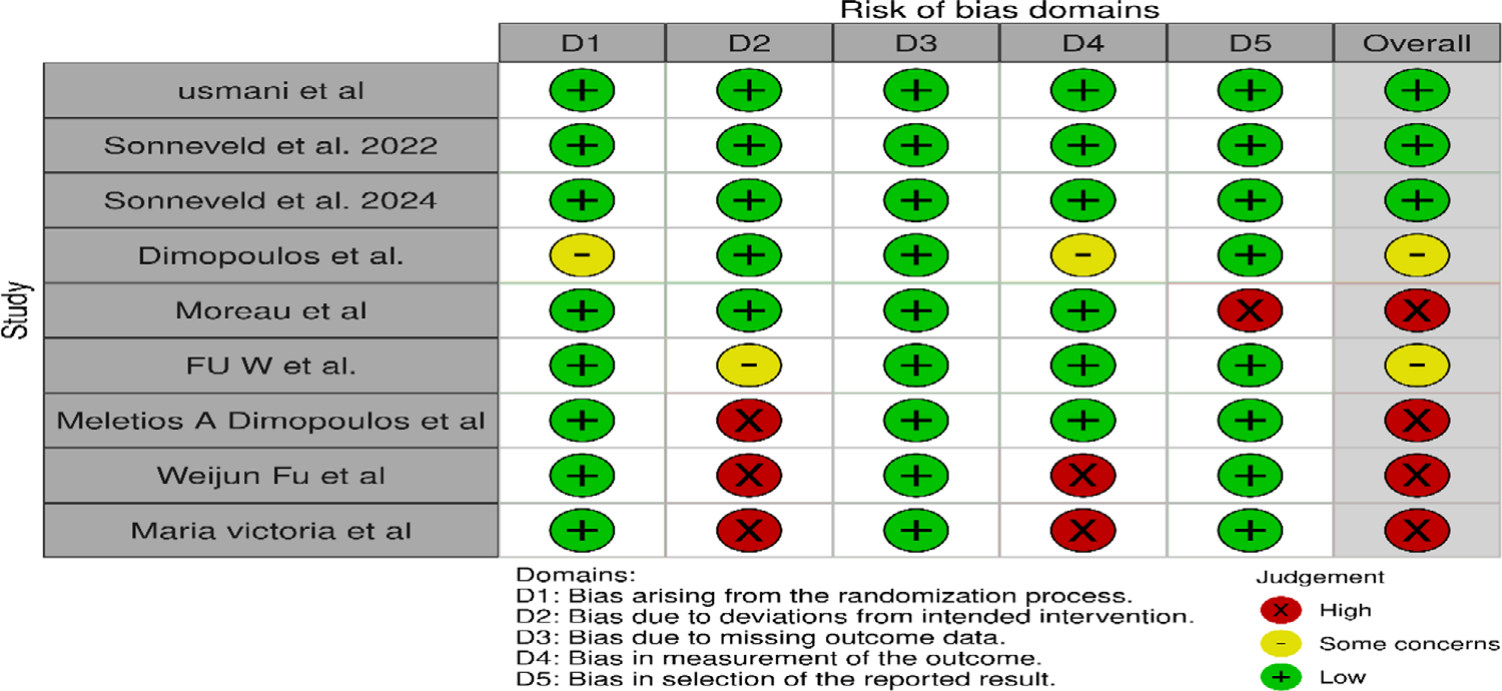
Risk of bias assessment of included studies.

Quality assessment and risk of bias were independently evaluated using the Cochrane Risk of Bias Tool 2.0 for all included RCTs. The assessment examines five key domains where bias could be introduced. These domains include bias from the randomization process, bias due to deviations from intended interventions, bias due to missing outcome data, bias in the measurement of outcome data, and bias in the selection of reported results. Each domain was carefully assessed and rated as having either a low risk of bias, some concerns, or a high risk of bias. Based on these individual evaluations, we then assigned an overall risk of bias judgement for each RCT to aid the interpretation of study reliability. The quality assessment was carried out by two independent investigators (H.A. and A.H.), and the results were compared. Any disagreements were resolved through discussion. A summary is provided in Figure 2 and Table S1.

### 2.5 Statistical Analysis

All statistical analyses were performed using Review Manager (Rev Man) software, version 5.4 (The Cochrane Collaboration, 2020). For the primary outcome of PFS, results were expressed as hazard ratios (HRs) with corresponding confidence intervals (CI). PFS was analyzed using the log HR and its standard error via the generic inverse variance method, with the HR serving as the main effect measure. For secondary outcomes, raw event data were analyzed using the Mantel–Haenszel method to calculate risk ratios (RRs). Forest plots were generated to represent the pooled results across studies visually. Heterogeneity among studies was assessed using Higgins’ *I* ^2^ statistic, with values below 50% considered acceptable. A *p*‐value of less than 0.05 was deemed statistically significant.

## 3 Results

Following an extensive literature search, nine Phase III Randomized Controlled Trials (RCTs) were included in this meta‐analysis, comparing daratumumab‐based regimens against standard therapy across multiple efficacy and safety endpoints. The analysis included a total of 4556 patients, with 2426 patients randomized to the daratumumab group and 2130 patients to the control group.

### 3.1 Progression‐Free Survival

**FIGURE 3:**
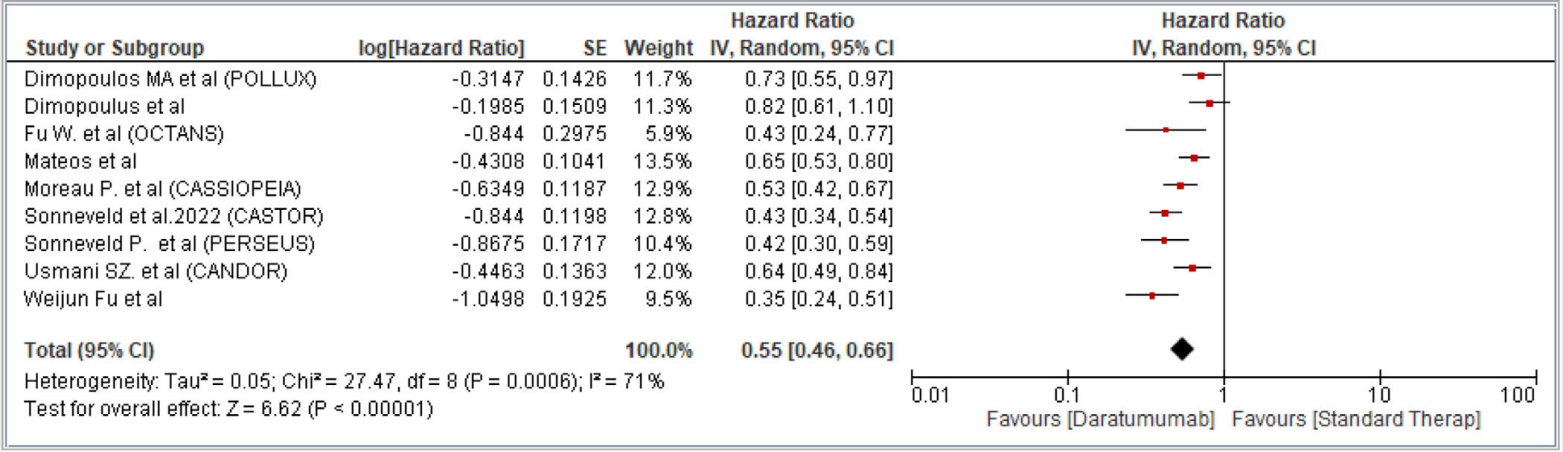
Forest plot of hazard ratios for progression‐free survival (PFS).

The pooled analysis demonstrated a statistically significant and clinically meaningful improvement in PFS for patients receiving daratumumab. The overall HR was 0.55 (95% CI: 0.46–0.66, *p* < 0.00001), indicating a 45% reduction in the risk of disease progression or death compared to the control group (Figure 3). This benefit was consistent across the included studies. Moderate heterogeneity was observed (*I* ^2^ = 71%). A leave‐one‐out sensitivity analysis confirmed the robustness of this finding, as the result remained statistically significant regardless of which individual study was omitted (pooled HR range: 0.52–0.58).

### 3.2 Overall Response Rate

**FIGURE 4:**
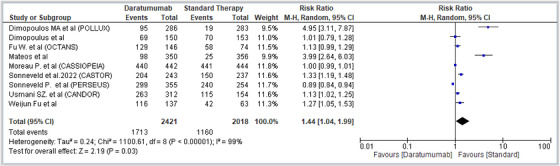
Forest plot of risk ratios for overall response rate (ORR).

All nine trials evaluated ORR. The pooled result showed a statistically significant improvement in ORR favoring the daratumumab group (RR = 1.44, 95% CI: 1.04–1.99, *p* = 0.03) (Figure 4). However, heterogeneity across the studies was exceptionally high (*I* ^2^ = 99%). Heterogeneity among studies was assessed using Higgins’ *I* ^2^ statistic, with values below 50% considered indicative of low‐to‐moderate heterogeneity. Statistical significance for pooled effect estimates was defined as *p* < 0.05. The leave‐one‐out sensitivity analysis revealed considerable instability in this pooled estimate, with results varying widely (RR range: 1.26–1.55) and losing statistical significance when most individual studies were omitted, reflecting substantial clinical or methodological variability between the trials.

### 3.3 Mortality

**FIGURE 5:**
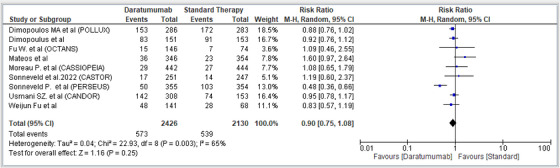
Forest plot of risk ratios for mortality outcomes.

Data from nine trials were pooled for OS. The analysis showed a non‐significant trend towards reduced mortality with daratumumab (RR = 0.90, 95% CI: 0.75–1.08, *p* = 0.25), with substantial heterogeneity (*I* ^2^ = 65%) (Figure 5).

A pre‐specified sensitivity analysis excluding the Sonneveld et al. (PERSEUS) trial substantially reduced heterogeneity (*I* ^2^ = 0%). Although the pooled estimate favored daratumumab (RR = 0.93, 95% CI: 0.85–1.02), the difference remained statistically non‐significant (*p* = 0.14). (Figure S1). This indicates that the overall finding is sensitive to the inclusion of specific study data.

### 3.4 Safety Outcomes

#### 3.4.1 Pneumonia

The incidence of pneumonia was significantly higher in the daratumumab group across all nine trials. The pooled RR was 1.74 (95% CI: 1.36–2.24, *p* < 0.0001), indicating a 74% increased relative risk. Heterogeneity was moderate (*I* ^2^ = 57%) (Figure S2).

#### 3.4.2 Secondary Malignancy

An analysis of eight trials demonstrated a statistically significant increased risk of secondary malignancies with daratumumab (RR = 1.46, 95% CI: 1.14–1.89, *p* = 0.003). The effect was consistent across the studies, with low heterogeneity (*I* ^2^ = 4%) (Figure S3).

#### 3.4.3 Hematological Adverse Effects

***Thrombocytopenia***: The pooled analysis of eight trials showed a statistically significant increased risk (RR = 1.17, 95% CI: 1.03–1.33, *p* = 0.01) with substantial heterogeneity (*I* ^2^ = 65%) (Figure S4). A sensitivity analysis omitting Mateos et al. strengthened this association and reduced heterogeneity (RR = 1.23, 95% CI: 1.11–1.36, *p* < 0.0001; *I* ^2^ = 31%) (Figure S5).

***Neutropenia***: Across nine trials, daratumumab was associated with a significantly higher risk of neutropenia (RR = 1.25, 95% CI: 1.07–1.46, *p* = 0.006). Heterogeneity was high (*I* ^2^ = 78%) (Figure S6). A leave‐one‐out sensitivity analysis was performed to assess the robustness of this finding and to investigate the source of the heterogeneity. The analysis demonstrated that the result was stable, with the pooled RR remaining statistically significant regardless of which individual study was omitted (RR range: 1.21–1.33). This confirms that the increased risk of neutropenia is a consistent and robust finding across the daratumumab clinical trial program.

***Lymphopenia***: The primary pooled analysis of seven trials showed a statistically significant increase in risk (RR = 1.38, 95% CI: 1.01–1.89, *p* = 0.04), with moderate heterogeneity (*I* ^2^ = 62%) (Figure S7). A sensitivity analysis removing the outlier study Sonneveld et al. [CASTOR] reduced heterogeneity and yielded a non‐significant result (RR = 1.14, 95% CI: 0.94–1.38, *p* = 0.17; *I* ^2^ = 8%) (Figure S8), indicating that a single trial drove the significant finding in the main analysis.

#### 3.4.4 Assessment of Publication Bias

We assessed the potential for publication bias for the primary outcome of PFS through visual inspection of a funnel plot (Figure S9). The funnel plot appeared roughly symmetrical, suggesting a low risk of publication bias. The funnel plot appeared roughly symmetrical, suggesting no obvious evidence of publication bias. However, visual inspection alone cannot exclude publication bias and should be interpreted cautiously given the limited number of included studies.

A funnel plot was not generated for the other outcomes, such as ORR, due to the high heterogeneity (*I* ^2^ = 99% in the case of ORR) and the known instability of the results, which would render the interpretation of a funnel plot unreliable. The extremely high heterogeneity observed for ORR (*I* ^2^ = 99%) likely reflects substantial clinical diversity among the included trials. Potential contributors include differences in disease status (newly diagnosed versus relapsed/refractory multiple myeloma), variation in backbone treatment regimens, transplant eligibility, duration of follow‐up, and differences between induction, consolidation, and maintenance treatment settings. These factors may have influenced response rates and should be considered when interpreting the pooled ORR estimate.

## 4 Discussion

This meta‐analysis and systematic review included a total sample size of 4556 patients, comparing daratumumab therapy with standard therapy in patients with malignancy. Main findings drawn from the nine Phase III RCTs indicated decreased risk of disease progression in the intervention group; however, decreased mortality with daratumumab‐based regimens has been reported in four out of nine RCTs, although they are not statistically significant. Moreover, the incidence of pneumonia was reported to be higher in the intervention group compared to the control group. Furthermore, secondary malignant events were evaluated to be significant in patients treated with Daratumumab, with higher risks in the CASTOR and CASSIOPEIA trials.

Treatment with daratumumab was initially approved as a treatment regimen for refractory/relapsed multiple myeloma; however, daratumumab has been reported to show enhanced efficacy with tolerable adverse effects [8]. In the recent CEPHEUS and AURIGA Phase 3 trial, outcomes, including PFS rate and disease progression rate, have been reported to be significantly reduced upon administering daratumumab with lenalidomide [9, 10]. Furthermore, multiple trials including CASSIOPEIA and GRIFFIN/PERSEUS Phase 3 trials have reported long‐term follow‐ups in patients with multiple myeloma, demonstrating alleviated PFS with lower risk in disease progression over longer periods [11, 12]. Previous meta‐analyses were unable to demonstrate positive outcomes with unavailable data as reported by Chang et al. [13, 14, 15]. Although a recent meta‐analysis written by Wang et al. collected data from the recently published trials, including ALCYONE and CASTOR trials, it reported data in patients with relapsed or refractory multiple myeloma, hence it was not as relevant [16, 17]. Recent studies reported improved health with no serious adverse effects, further emphasizing the likelihood of daratumumab as a potential treatment in the elderly suffering from multiple myeloma. However, data regarding the administration of the daratumumab combination in the broad population is not available yet [18].

Our analysis observed significant results that were reported in the five trials, including POLLUX, CANDOR, LEPUS, ALCYONE, and CASTOR, which favored daratumumab administration, evaluating improved PFS and ORR. Several studies conducted analyses and reported outcomes upon administration of daratumumab. A systematic review and meta‐analysis conducted reported the efficacy of daratumumab in patients with relapsed or refractory multiple myeloma, observing similar outcomes [7, 18]. Another systematic review and meta‐analysis conducted evaluated improved PFS with increased ORR and very good partial response (VGPR). The study also reported an increased OS rate with prolonged life and improved remission rate with reduced circumstances of death in patients treated with various regimens of daratumumab [19]. However, similar to our results, some meta‐analyses that were previously conducted also reported increased prevalence of neutropenia and thrombocytopenia, emphasizing on hematological side effects of daratumumab administration [18, 19]. In addition, increased risks of diarrhea, upper respiratory tract infection, and pneumonia were also observed [20]. Our systematic review and meta‐analysis focused on the efficacy of daratumumab in patients with multiple myeloma; however, previous meta‐analyses primarily focused on relapsed/refractory multiple myeloma populations, whereas the present study incorporates evidence from both relapsed/refractory and newly diagnosed treatment settings, providing a broader assessment of daratumumab efficacy and safety.

Our analysis included the PERSUS trial, which favored standard therapy over daratumumab. Overall heterogeneity was high in terms of ORR and mortality whereas in terms of PFS it was moderate. CASTOR and CASSIOPEIA trials reported in our meta‐analyses also demonstrated secondary malignant events with a very high risk.

Although there isn't enough evidence supporting the clinical use of daratumumab in treating patients with multiple myeloma, but our results report its efficacy and safety with low risks being observed.

To explore the sources of heterogeneity observed in some outcomes, we conducted both subgroup and leave‐one‐out (LOO) sensitivity analyses. Sub‐group analysis showed consistent results in POLLUX and CASTOR trials; however, CANDOR and PERSEUS trials showed variability due to different regimen choices. In the case of ORR and mortality, exclusion of the PERSEUS trial lowered heterogeneity, hence favoring daratumumab. Similarly, exclusion of the CASSIOPEIA trial lowered the risk of secondary malignancy events. These variations are most likely due to different daratumumab regimens, follow‐up durations, and long‐term updates from CANDOR and CASSIOPEIA. Despite variations, the exclusion of certain trials improved the ORR, PFS, and mortality, which emphasized the efficacy and safety of daratumumab.

### 4.1 Limitations of the Included Evidence

First, the high statistical heterogeneity observed in the primary analysis, although explored and resolved through sensitivity analysis, indicates underlying clinical diversity in the study designs of trials.

A consistent issue across all studies was the open‐label design, which introduces the possibility of reporting bias. In addition, most primary analyses were based on relatively short follow‐up periods, resulting in immature OS data for studies such as POLLUX, CANDOR, and PERSEUS. Consequently, it remains uncertain whether the PFS benefits observed will translate into long‐term survival advantages or sustained improvements in quality of life. Moreover, some studies, including CASSIOPEIA and PERSEUS, restricted entry to a comparatively younger, transplant‐eligible cohort. Similarly, differences in access to supportive care, symptom management, post‐progression therapies, and prescribing practices within healthcare systems would have affected long‐term outcomes. This selective patient population, combined with loss to follow‐up during extended observation periods, diminishes external validity and limits applicability to the broader myeloma population. In addition, trials with negative results are less likely to be reported than those with positive outcomes. Despite being investigated and reduced by sensitivity analyses, the high statistical heterogeneity found in the primary analysis most likely reflects underlying clinical diversity among the included studies. This variability was caused by differences in the doses given, the duration of follow‐up periods, and the participants' previous treatments.

### 4.2 Limitations of the Review Process

Several limitations of our methodology should be acknowledged. The small number of studies included precluded a formal assessment of publication bias using funnel plots, as such tests are known to be underpowered with fewer than 10 studies. Although a funnel plot was generated and visually inspected for the primary outcome of PFS, the relatively small number of studies limited the reliability of formal statistical tests for funnel plot asymmetry such as Egger's or Begg's tests. Furthermore, our search was restricted to published literature in major databases, which may have omitted relevant grey literature or studies in non‐English journals, potentially introducing a selection bias. In addition, trials with negative results are less likely to be reported than those with positive outcomes.

## 5 Conclusions

This meta‐analysis of nine Phase III RCTs showed that daratumumab‐based regimens considerably increase PFS in multiple myeloma, but the OS advantage has yet to be proven. While efficacy is constant across trials, varied response rates and heterogeneity lead to disparities in outcomes. Importantly, therapy increases the risk of pneumonia, secondary malignancies, and hematologic toxicities, which must be closely monitored. Sensitivity analysis indicated that trial‐specific changes in regimens and follow‐up account for the majority of the heterogeneity observed. Overall, daratumumab is a highly successful therapeutic option for slowing disease development, but its use should be adjusted to individual patient profiles, with careful consideration of safety hazards.

## Author Contributions

**AM and MK** contributed in making the search strategy, retrieving results from databases, performing data extraction and analysis, resolving conflicts during screening and risk of bias assessment, and rewriting the Introduction and revising the Methodology section. **HA** contributed in screening, data extraction, and preparing the baseline characteristics section of the manuscript. **US and WBK** performed the risk of bias analysis, contributed in grading the evidence, and assisted in writing the manuscript. **HA and TF** contributed in writing the initial draft of the Introduction and the Materials and Methods section of the manuscript. **ASA and SF** contributed in writing the study setting, lesion characteristics, and baseline parts in the Results section of the manuscript. **MHH** contributed in writing the Results of outcomes section, and verified statistical consistency. **MK** contributed in screening and data extraction, discussion writing and revising the article. **H.A** prepared the funnel plots and conducted the publication bias analysis. **TF** provided supervision, project administration, final review, and editing of the manuscript. All authors read and approved the final version of the manuscript.

## Funding

The authors have nothing to report.

## Ethics Statement

The authors have nothing to report.

## Consent

The authors have nothing to report.

## Conflicts of Interest

The authors declare no conflicts of interest.

## Supporting information




**Supporting File 1**: jha270362‐sup‐0001‐FigureS1.docx

**Supporting File 2**: jha270362‐sup‐0002‐FigureS2.docx

**Supporting File 3**: jha270362‐sup‐0003‐FigureS3.docx

**Supporting File 4**: jha270362‐sup‐0004‐FigureS4.docx

**Supporting File 5**: jha270362‐sup‐0005‐FigureS5.docx

**Supporting File 6**: jha270362‐sup‐0006‐FigureS6.docx

**Supporting File 7**: jha270362‐sup‐0007‐FigureS7.docx

**Supporting File 8**: jha270362‐sup‐0008‐FigureS8.docx

**Supporting File 9**: jha270362‐sup‐0009‐FigureS9.docx

**Supporting File 10**: jha270362‐sup‐0010‐FigureS10.docx

## Data Availability

All data generated or analyzed during this study are included in this published article and its supplementary information files. The extracted data supporting the findings of this meta‐analysis can be requested from the corresponding author on reasonable request.
