# Supplementary figures and images for: Efficacy and Safety of Daratumumab‐Based Regimens in Multiple Myeloma: A Systematic Review and Meta‐Analysis of Phase III Randomized Controlled Trials
Source: EJHaem. 2026 Jul 25;7(4):e70362. doi: 10.1002/jha2.70362 (PMC13401138; doi:10.1002/jha2.70362)

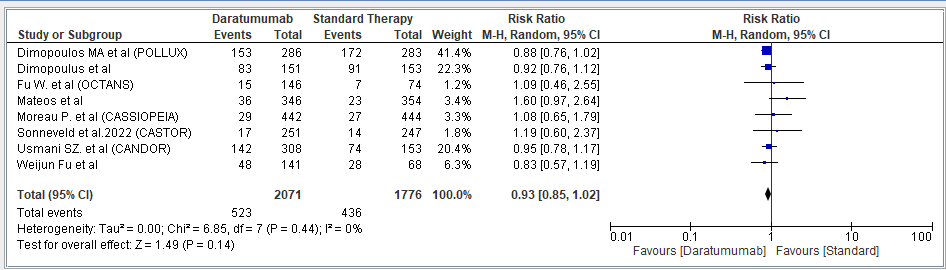


Supplementary Figure 1: Sensitivity analysis Mortality without Sonneveld et al. (PERSEUS).

Supplement: Supplementary file 1 — Supporting File 1: jha270362‐sup‐0001‐FigureS1.docx [file JHA2-7-e70362-s002.docx]

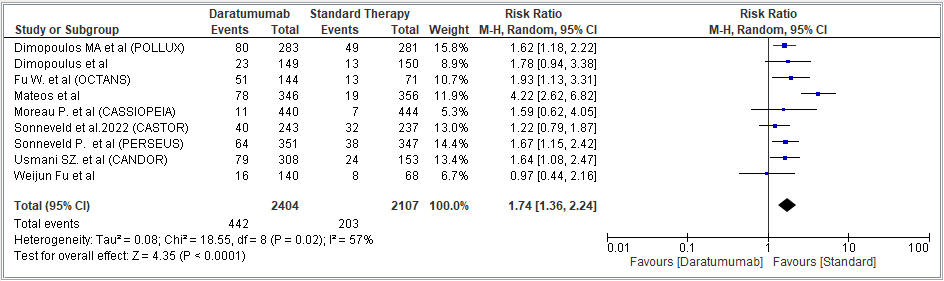


Supplementary Figure 2: Forest Plot of Risk Ratios for Pneumonia.

Supplement: Supplementary file 2 — Supporting File 2: jha270362‐sup‐0002‐FigureS2.docx [file JHA2-7-e70362-s008.docx]

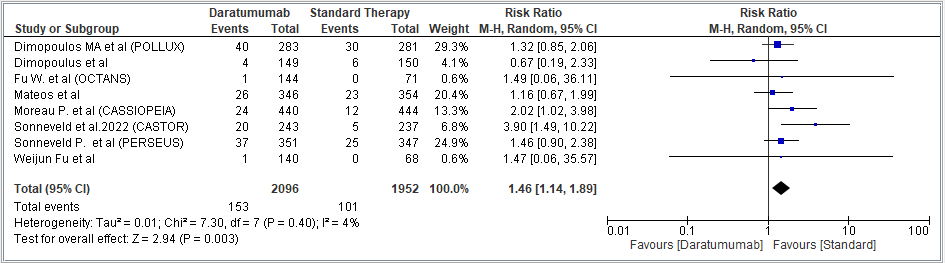


Supplementary Figure 3: Forest Plot of Risk Ratios for Secondary Malignancies.

Supplement: Supplementary file 3 — Supporting File 3: jha270362‐sup‐0003‐FigureS3.docx [file JHA2-7-e70362-s009.docx]

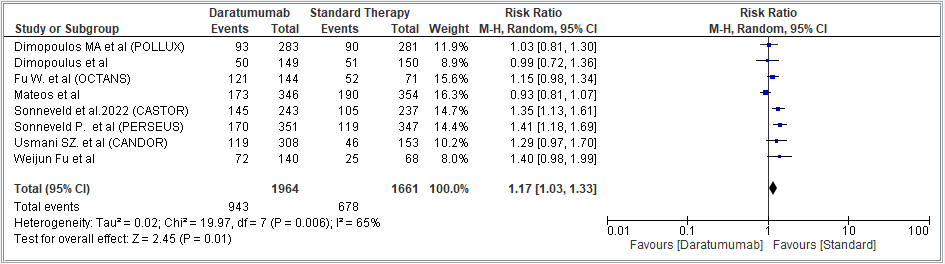


Supplementary Figure 4: Forest Plot of Risk Ratios for Thrombocytopenia.

Supplement: Supplementary file 4 — Supporting File 4: jha270362‐sup‐0004‐FigureS4.docx [file JHA2-7-e70362-s010.docx]

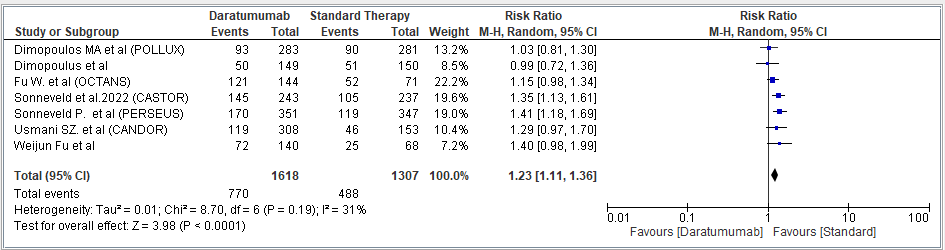


Supplementary Figure 5: Sensitivity analysis Thrombocytopenia without Mateos et al. (ALCYONE).

Supplement: Supplementary file 5 — Supporting File 5: jha270362‐sup‐0005‐FigureS5.docx [file JHA2-7-e70362-s003.docx]

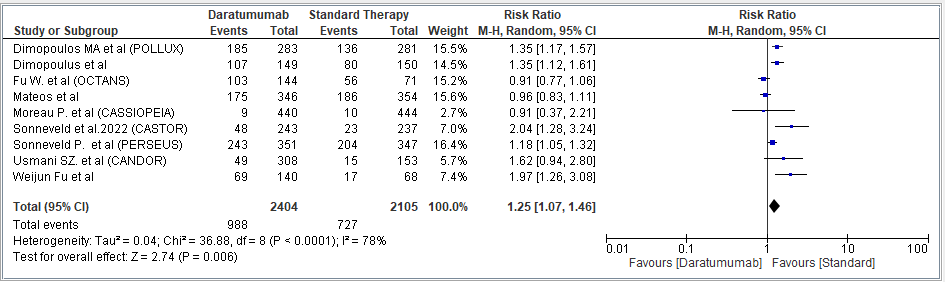


Supplementary Figure 6: Forest Plot of Risk Ratios for Neutropenia.

Supplement: Supplementary file 6 — Supporting File 6: jha270362‐sup‐0006‐FigureS6.docx [file JHA2-7-e70362-s006.docx]

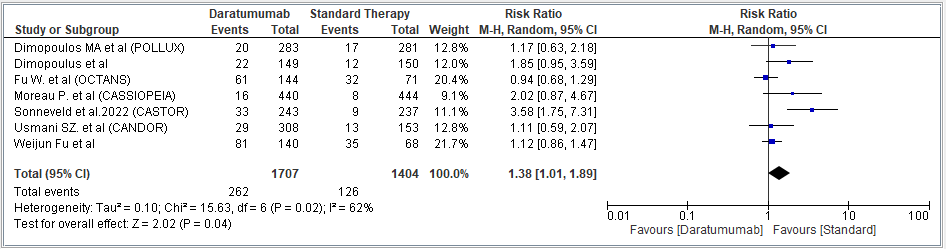


Supplementary Figure 7: Forest Plot of Risk Ratios for Lymphocytopenia.

Supplement: Supplementary file 7 — Supporting File 7: jha270362‐sup‐0007‐FigureS7.docx [file JHA2-7-e70362-s001.docx]

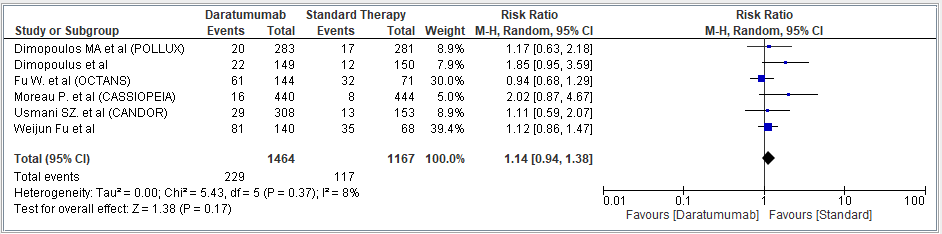


Supplementary Figure 8: Sensitivity Analysis Lymphocytopenia without Sonneveld et al. [CASTOR].

Supplement: Supplementary file 8 — Supporting File 8: jha270362‐sup‐0008‐FigureS8.docx [file JHA2-7-e70362-s005.docx]
