# Supplementary material for: Efficacy and Safety of Daratumumab‐Based Regimens in Multiple Myeloma: A Systematic Review and Meta‐Analysis of Phase III Randomized Controlled Trials
Source: EJHaem. 2026 Jul 25;7(4):e70362. doi: 10.1002/jha2.70362 (PMC13401138; doi:10.1002/jha2.70362)
Supplement: Supplementary file 9 — Supporting File 9: jha270362‐sup‐0009‐FigureS9.docx [file JHA2-7-e70362-s007.docx]

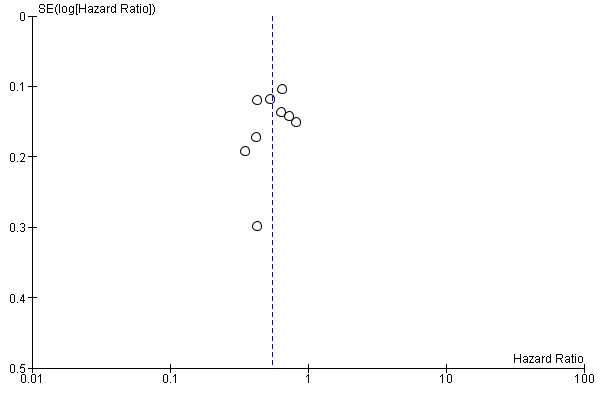


Supplementary Figure 9: Publication bias funnel plot for the primary outcome of Progression-Free Survival (PFS).
