# Supplementary material for: Efficacy and Safety of Daratumumab‐Based Regimens in Multiple Myeloma: A Systematic Review and Meta‐Analysis of Phase III Randomized Controlled Trials
Source: EJHaem. 2026 Jul 25;7(4):e70362. doi: 10.1002/jha2.70362 (PMC13401138; doi:10.1002/jha2.70362)
Supplement: Supplementary file 10 — Supporting File 10: jha270362‐sup‐0010‐FigureS10.docx [file JHA2-7-e70362-s004.docx]

| Study | D1 | D2 | D3 | D4 | D5 | Overall |
| --- | --- | --- | --- | --- | --- | --- |
| usmani et al | Low | Low | Low | Low | Low | Low |
| Sonneveld et al. 2022 | Low | Low | Low | Low | Low | Low |
| Sonneveld et al. 2024 | Low | Low | Low | Low | Low | Low |
| Dimopoulos et al. | Some concerns | Low | Low | Some concerns | Low | Some concerns |
| Moreau et al | Low | Low | Low | Low | High | High |
| FU W et al. | Low | Some concerns | Low | Low | Low | Some concerns |
| Meletios A Dimopoulos et al | Low | High | Low | Low | Low | High |
| Weijun Fu et al | Low | High | Low | High | Low | High |
| Maria victoria et al | Low | High | Low | High | Low | High |

Supplementary Table 1: Risk of Bias Assessment of Included Studies.
